# Over-eruption in marsupial carnivore teeth: compensation for a constraint

**DOI:** 10.1098/rspb.2023.0644

**Published:** 2023-12-13

**Authors:** Menna E. Jones

**Affiliations:** School of Natural Sciences, University of Tasmania, Hobart, Tasmania 7001, Australia

**Keywords:** mammalian carnivore, Dasyuridae, Thylacinidae, tooth eruption, evolutionary constraint, functional tooth replacement

## Abstract

Pronounced over-eruption of the canine teeth, causing the cervical enamel margin to extend beyond the alveolar bone and exposing the root, occurs with age and growth in Australian marsupial carnivores, much more than in eco-morphologically equivalent placental carnivores. Suppression of functional tooth replacement is characteristic of marsupials, where most placentals have the primitive diphyodont pattern of two generations of incisor, canine and premolar teeth. Canine and molar tooth dimensions of four species of marsupial carnivores (thylacine *Thylacinus cynocephalus*, Tasmanian devil *Sarcophilus harrisii* and two quolls *Dasyurus* spp*.*) and canine dimensions of seven eco-morphologically equivalent placental carnivore species were measured from museum specimens. Canine dimensions were measured in a time series on live wild-living individual devils and quolls. The canine teeth and to a lesser extent the molar teeth of marsupial carnivores continue to erupt through life, resulting in a net increase in tooth height and diameter, a phenomenon not evident in placental carnivores. Potential mechanisms causing over-eruption include tooth wear and gradual release of occlusal pressure as the individual grows. Over-eruption in marsupial carnivores may be a compensatory response for tooth size limits imposed by monophyodont tooth replacement, ensuring that animal's teeth are scaled to jaw size from juvenile to adulthood.

## 1. Introduction

**Figure 1.**
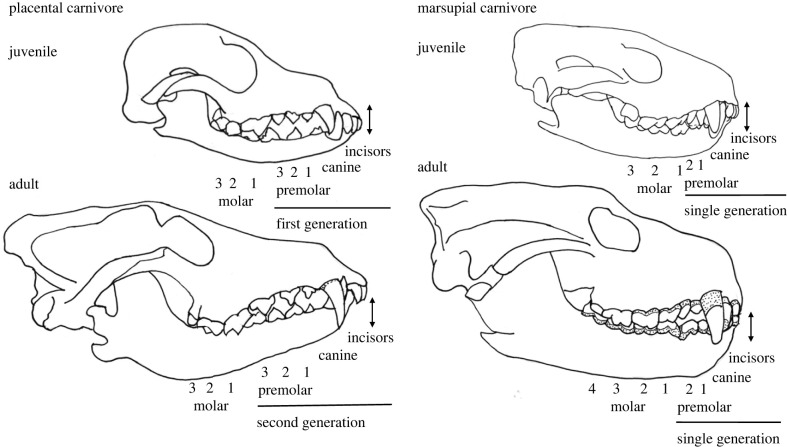
Differences in dental eruption sequences between placental and marsupial carnivores illustrated with representative skulls of a weaned juvenile and an adult placental dingo (*Canis dingo*) and marsupial Tasmanian devil (*Sarcophilus harrisii*). Placental carnivores have two generations of anterior teeth (incisors, canines and premolars), scaled to the size of the juvenile and adult jaw, respectively. Marsupial carnivores have a single generation of anterior teeth that are fully erupted and functional at weaning in a small juvenile jaw. The single generation of molar teeth in both taxa erupt in sequence from anterior to posterior as the jaw grows in length. The same-sized double-headed arrows demonstrate how the second-generation canine teeth in adult placental carnivores are larger than the first-generation canines in juveniles, and how over-eruption in marsupial carnivores results in larger (taller and wider) canine teeth and taller molar (as well as incisor and premolar) teeth in adults compared with juveniles. Shaded areas of teeth in both marsupial and placental carnivores are where root is exposed by over-eruption.

There has long been interest in comparing the biology of placental and marsupial mammals, and how phylogenetically distinct traits relate to adaptations and convergent eco-morphological niches in different parts of the world [e.g. 1,2]. One trait of interest is the ontogenetic pattern of anterior tooth replacement, with marsupials and some placental mammals exhibiting the monophyodont condition and most placentals the primitive therian diphyodont condition (figure 1). The mammalian pattern of two generations of anterior dentition is considered to have evolved in response to the rapid determinant growth associated with lactation [3]. Teeth are not required until weaning when a set of small first generation teeth scaled to the size of the juvenile jaw are fully erupted, to be replaced by a second generation, permanent set of larger incisors, canines and premolars as the animal approaches its mature body size. Molar teeth are not replaced, in either the monophyodont or diphyodont condition, as these erupt sequentially at the rear of the tooth row as the animal grows (figure 1) [3–5]. Suppression of the first generation of anterior teeth is characteristic of marsupial mammals [6], including the carnivorous marsupials in the Family Dasyuridae [7,8], and fairly common among diverse taxa of placental mammals [3]. In marsupials, the first generation incisors and canines are vestigial and un-erupted, and the first two premolars are developmentally retarded with no evidence of second generation replacements [6,8]. Post-birth marsupial teat attachment has been proposed as an explanation for mechanical suppression of odontogenesis in marsupials [4], but suppression of tooth replacement in placental mammals begs alternative explanations [3].

In the monophyodont Australian marsupial carnivores (Order Dasyuromorphia; Families Thylacinidae, Dasyuridae), the single set of anterior teeth, which must serve the animal as a fully grown adult, erupt fully into a jaw that is much too small to accommodate adult-sized teeth (figure 1) [3,4]. The question then is how the monophyodont pattern of tooth replacement in marsupial carnivores affects ontogenetic dental function. It might operate as a phylogenetic constraint if the single set of anterior teeth, which are fully erupted and functional at weaning, are smaller relative to jaw size in adults than the second generation teeth of eco-morphologically equivalent adult placental carnivores. Functional dentition is required at weaning and large canine teeth are fundamental to killing and feeding behaviour in all predatory mammalian carnivores. Direct comparisons of canine tooth measurements between marsupial and placental carnivores are complicated, however, by canine shape, which influences tooth function and varies with diet and killing and hunting type, and so is confounded with phylogeny [2,9–11]. Werdelin [4] suggested that the marsupial pattern of tooth replacement does impose a constraint on molar tooth and jaw morphology during ontogeny and could explain the greater evolutionary plasticity in placental compared with marsupial carnivores.

**Figure 2.**
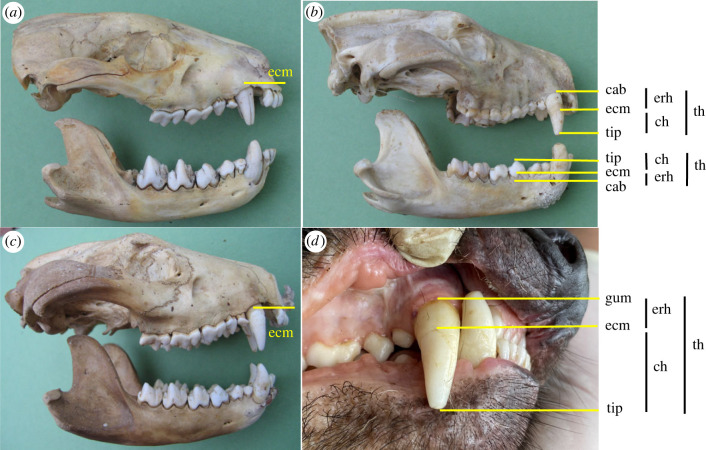
Images of the teeth of Tasmanian devils showing canine and molar tooth measurements, and over-eruption and wear in canine and molar teeth at different ages. (*a*) Weaned juvenile: molars M1 to M3 fully erupted, lower M4 one cusp erupting, tooth wear minimal (score 1 on canines and M1, no wear M2, M3). (*b*) Young adult (almost 2 years old): molar eruption complete, small amount of over-eruption on canines, tooth wear minimal (score 1 on canines and M2, no wear M1, M3–M4. (*c*) Older adult (approx. 4 years old): pronounced over-eruption of canine (enamel cervical margin half-way down the tooth), substantial tooth wear (scores of 2 upper canine, lower canine broken in life with wear facet on posterior edge, scores of 1, 3, 3, 2 for M1-M4. (*d*) Two-year-old live wild male devil with significant canine over-eruption and wear (score 2) on the canine. *a*–*c* From museum specimens; *d* wild devil (photo: David Hamilton). cab, crest of alveolar bone; gum, gum line; ecm, enamel cervical margin; erh, exposed root height; tip, crown tip; ch, crown height; th, total height.

Pronounced over-eruption, greater than observed in eco-morphologically similar placental carnivores (figures 1 and 2), is evident in the canine teeth of all extant and recently extinct marsupial carnivore species but has not been studied. Over-eruption is the physiological movement of a tooth out of the line of occlusion, resulting in the enamel cervical margin of the tooth crown extending beyond the level of the alveolar bone and exposing the root [12]. Tooth over-eruption in mammals, both human and wild non-human, is reported to be a response to both tooth wear and/or a lack of an occlusal partner to the over-erupted tooth [13–15]. The effects of these mechanisms are not mutually exclusive. If tooth wear is a factor, teeth with occlusal partners (molar teeth) should maintain a similar height as tooth wear progresses. Teeth without complete occlusion (canine teeth) might be expected to increase in height and over-erupt to a greater extent, both in response to tooth wear and gradually releasing oblique occlusal pressure as the animal grows.

In this paper, I explore whether over-eruption of the single generation (second-generation, unreplaced) canine teeth in marsupial carnivores could function to compensate in adulthood for the constraint of comparatively smaller teeth erupting into a small juvenile jaw, a consequence of suppression of anterior tooth replacement. I compare the amount of canine over-eruption between the monophyodont Australian marsupial carnivores and eco-morphologically equivalent diphyodont placental carnivores, in which the second-generation permanent canines erupt into a nearly full-grown jaw. In marsupial carnivores, I also measure age-related changes in canine dimensions through repeat measurement on live individuals in a wild population, and size-related differences in canine and molar tooth dimensions from skulls.

I consider Australian carnivorous marsupial taxa in the Order Dasyuromorphia: the thylacine (*Thylacinus cynocephalus*, Family Thylacinidae), and the Tasmanian devil (*Sarcophilus harrisii*) and species of quolls (spotted-tailed quoll *Dasyurus maculatus*, eastern quoll *D. viverrinus*) in the Family Dasyuridae. In the placental Order Carnivora, I measure seven species, all native to Israel, that represent the diversity of placental families that are eco-morphologically equivalent to the Australian marsupial carnivore species [2]. Analogues are canid-like: thylacine versus the larger grey wolf (*Canis lupus*) and smaller golden jackal (*C. aureus*) (F. Canidae); specialist bone eaters: devil versus larger striped hyaena (*Hyaena hyaena*, F. Hyaenidae) and similar-sized but non-osteophageous Caucasian badger (*Meles canescens*, F. Mustelidae) [2,16]; small carnivores that are eco-morphological analogues of the marsupial quolls: the Egyptian mongoose (*Herpestes ichneumon*, F. Herpestidae), stone marten (*Martes foina*) and the marbled polecat (*Vormela peregusna*) both in the Family Mustelidae [2].

## 2. Methods

Tooth and skull measurements were taken using Vernier calipers (0.01 mm accuracy) from 15 to 124 individuals per species of wild-caught museum specimens of four marsupial and seven placental carnivores (tables 1 and 3), and tooth measurements from 20 to 153 individuals per species of three species of live wild-living marsupial carnivores: devils, spotted-tailed quolls and eastern quolls. Australian marsupial carnivores were represented by specimens housed in collections of the Tasmanian Museum and Art Gallery (Hobart, Tasmania), Queen Victoria Museum (Launceston, Tasmania), Museum of Victoria (Melbourne, Victoria), Donald Thomson and Department of Fisheries and Wildlife Collections at the Museum of Victoria, Australian Museum (Sydney, New South Wales (NSW)). Museum specimens of placental carnivores were measured in the collection of the Tel Aviv Museum of Natural History at Tel Aviv University in Israel. Tooth measurements were taken from live, wild-living marsupial carnivores multiple times over their lifetime. All individuals were of known age because they had been first trapped as young animals. Lifespans are short (Tasmanian devil: 6 years, spotted-tailed quoll: 4 years, eastern quoll: 3 years) and devils and quolls can be accurately aged by body size, tooth eruption and tooth wear up to 3 years of age. All measurements were taken in healthy populations, free from devil facial tumour disease (DFTD), during fieldwork at Cradle Mountain National Park in Tasmania, Australia, between 1990 and 1993 (field methods in: [17]).

**Table 1.** The relationship between the amount of canine tooth over-eruption and skull length (CBL) in marsupial and placental carnivores with predicted values of over-eruption for the smallest and largest individual of each species in the dataset. The ‘estimate' is the slope of the regression for each species. CBL and extent of over-eruption are measured in mm. Subclasses: M, marsupial; P, placental. *N*, number of individuals. CBL, condylobasal length measured on the skull. s.e., standard error.

subclass	scientific name	common name	*N*	slope ± s.e.	*p-*value	smallest CBL	predicted over-eruption ± s.e.	largest CBL	predicted over-eruption ± s.e.
M	*Sarcophilus harrisii*	Tasmanian devil	20	0.476 ± 0.080	<0.0001	116.2	2.40 ± 0.96	139.1	13.32 ± 1.20
P	*Hyaena hyaena*	Striped hyaena	29	0.014 ± 0.006	0.05	200.0	0.40 ± 0.12	235.0	0.89 ± 0.15
P	*Canis lupus*	Grey wolf	43	−0.004 ± 0.002	0.06	225.0	0.17 ± 0.06	180.0	0.37 ± 0.06
M	*Thylacinus cynocephalus*	Thylacine	13	0.065 ± 0.036	0.11	184.9	4.18 ± 1.58	252.7	8.56 ± 1.39
M	*Dasyurus maculatus*	Spotted-tailed quoll	19	0.096 ± 0.058	0.13	89.2	2.33 ± 0.90	115.0	4.80 ± 0.96
P	*Herpestes ichneumon*	Egyptian mongoose	18	−0.006 ± 0.004	0.16	102.8	0.10 ± 0.03	84.4	0.21 ± 0.06
P	*Vormela peregusna*	Marbled polecat	28	0.006 ± 0.004	0.15	46.7	0.03 ± 0.02	50.9	0.07 ± 0.02
P	*Canis aureus*	Golden jackal	34	0.003 ± 0.003	0.22	143.0	0.07 ± 0.04	168.0	0.16 ± 0.04
M	*Dasyurus viverrinus*	Eastern quoll	20	0.042 ± 0.046	0.38	69.4	1.22 ± 0.45	89.7	2.06 ± 0.61
P	*Meles canescens*	Caucasian badger	33	0.004 ± 0.006	0.45	114.4	0.07 ± 0.04	131.9	0.15 ± 0.07
P	*Martes foina*	Stone marten	15	0.014 ± 0.030	0.65	74.3	0.19 ± 0.14	82.6	0.31 ± 0.15

**Table 3.** Coefficients for the linear trends of the relationship between a) molar over-eruption and b) molar height and jaw length for each tooth, M1 to M4, across the molar tooth row for marsupial carnivores in museum collections. s.e., standard error; *t*, t test; *p*, *p*-value; *R*_adj_, adjusted *r*^2^; *N*, sample size.

species	linear trend	s.e.	t	*p*-value	*R*_adj_	*N*
(*a*) molar over-eruption and jaw length						
*D. viverrinus*	−0.02363	0.013506	−1.74994	0.088649	0.569291	40
*D. maculatus*	−0.00545	0.009697	−0.56169	0.577805	0.598754	40
*S. harrisii*	−0.02982	0.009307	−3.20343	0.001754	0.724293	124
*T. cynocephalus*	−0.01076	0.004421	−2.43459	0.017135	0.821568	88
(*b*) molar height and jaw length						
*D. viverrinus*	−0.04126	0.018342	−2.24947	0.030692	0.183943	40
*D. maculatus*	−0.02828	0.015788	−1.79096	0.082206	0.280759	38
*S. harrisii*	−0.00637	0.015476	−0.41187	0.68127	0.477064	114
*T. cynocephalus*	0.02278	0.007481	3.045123	0.003195	0.830572	84

### (a) Measurements

Tooth measurements were defined as follows (and figure 2): 1) canines. Total tooth height (TH) was measured on skulls from the midline of the crest of the alveolar bone to the canine tip. Bone resorption that eroded the alveolar crest was observed on a few skulls but the position of the original alveolar crest could be clearly estimated from adjacent bone. On live animals, total tooth height was measured from the midline of the crest of the gum or from a clear brown stain indicating the previous position of the gum, in cases of gum recession, to the canine tip. Crown height was measured from the midline or crest of the enamel cervical margin (the edge of the enamel that covers the crown of the tooth) to the tip. Maximum anteroposterior (APD) and mediolateral (MLD) diameters corresponded to the diameters at the level of the crest of the alveolar bone (skulls) or gum line (live animals). 2) Molars. Total height was measured from the crest of the alveolar bone to the tip of the tallest cusp; crown height from the crest of the enamel cervical margin to the tip of the tallest cusp by holding the calipers level with the margin and crest on both anterior and posterior roots simultaneously. Extent of over-eruption for all teeth, defined as the height of exposed root above the dentine-enamel margin, was calculated for both canines and molars as the difference between total tooth and crown height. I follow the commonly used nomenclature for marsupial molar teeth (M1–M4) [18].

One upper canine tooth and the lower molar row (M1–M4) on one side of the jaw were measured on each marsupial skull or wild animal and one upper canine tooth on the skulls of placentals. A wear stage was assigned to all measured teeth in marsupial carnivores using schemes devised for canines of marsupial carnivores [19] and for lower molars of devils [20] as follows: 0 = no wear, tip or cusp sharp; 1 = tip or cusp worn, no dentine exposed; 2 = tip or cusp worn, dentine exposed; 3 = tip or cusp well worn, dentine exposed, less than half tooth height worn; 4 = tip or cusp well worn, dentine exposed, more than half tooth height worn; and 5 = tooth worn to gum.

Body size was measured on skulls as condylobasal length (CBL) (from the base of the first upper incisor [I_1_] to the tip of the occiput) and jaw length (from the base of the first lower incisor [I_1_] to the mandibular condyle). Jaw length was used in analyses where the high incidence of broken skulls markedly reduced sample sizes, as mandibles have lower incidents of breakage.

All substantially undamaged skulls in museum collections were measured, with teeth broken in life (some wear on the broken edge) or following death (sharp edges to the break) excluded from measurement. Individuals of a range of ages and tooth wear stages were measured, from independent juveniles to fully grown adults (full dental eruption), with small (dependent) juveniles excluded based on incomplete eruption of M2 in marsupials and incomplete dental eruption and open sutures in placentals. Total and crown height of canine teeth were measured in both marsupial and placental carnivores. For marsupial carnivores, the two maximum diameters of canine teeth (in the anteroposterior and mediolateral planes), as well as total height and crown height of molar teeth were also measured. All four teeth in the molar row were measured for devils and thylacines, and just M1 and M4 for spotted-tailed and eastern quolls.

For wild-living devils, spotted-tailed and eastern quolls, total tooth height, crown height and the two maximum diameters of the canine teeth were measured repeatedly during growth on individuals trapped over a 2.5-year period. The individuals included a range of starting ages (independent juvenile to adult) with definitive ages known for most individuals in this population as they were first trapped as young animals.

### (b) Sample sizes

Skulls and canine teeth for the comparison of canine over-eruption between marsupial and placental carnivores were measured from between 13 and 43 individuals per species in museum collections (table 1). For analysis of canine tooth eruption and dimensions in marsupial carnivores, the number of different individuals measured on museum specimens and wild animals were: thylacine (23 museum, 0 wild), Tasmanian devil (33 museum, 153 wild individuals with repeat measurements through time of canine teeth), spotted-tailed quoll (20 museum, 19 wild), eastern quoll (20 museum, 61 wild). Sample sizes for analysis of over-eruption and height of molar teeth from museum specimens were: both quoll species (40), devils (124) and thylacines (88) (table 3). The much higher natural and post-mortem fracture rates of elongate canine teeth compared with more rounded molar teeth account for the discrepancy in sample sizes from museum specimens between canine and molar teeth. Variation in sample sizes among different measures reflect tooth breakage or loss in individual live or museum specimens.

### (c) Statistical analyses

The relationship between the degree of canine over-eruption and body size across marsupial and placental carnivore species in museum collections was analysed using generalized linear models (GLM), with a Gaussian distribution and species and log(CBL) as covariates. Changes in canine tooth dimensions over time in wild marsupial carnivores were analysed using a generalized linear mixed model using a likelihood ratio test (LRT, χ^2^) to test for significance. Species comparisons among marsupial carnivores in the changes in three canine dimensions in museum specimens were analysed using a GLM with Gaussian distribution and species and jaw length as covariates. To compare the trends of over-eruption or height across the four-tooth molar row (from M1 to M4) with body size among species, the many-model function in the Broom package was used (https://r4ds.had.co.nz/many-models.html; accessed 23 December 2022). These models were fit with linear, quadratic and cubic trend functions. Throughout this paper, *n* is the number of observations, and $r_{\mathrm{adj}}^{2}$ is the adjusted squared multiple *r* (correlation coefficient). All analyses were conducted in the R Project for Statistical Computing (version 4.2.2, 2022.10.31) using R Studio (version 2022.12.0).

## 3. Results

### (a) Over-eruption of canine teeth

**Figure 3.**
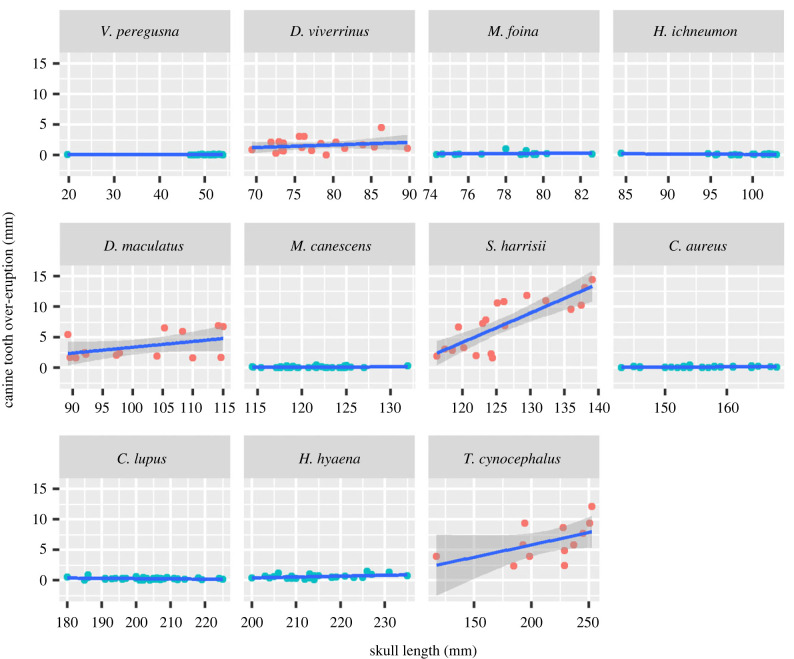
The extent of over-eruption of the canine teeth plotted against skull length in adult individuals of four species of marsupial carnivores and seven species of similar-sized and ecologically similar placental carnivores held in museum collections. Species are presented left to right, top to bottom, from smallest to largest skull size. Red symbols are marsupial species and blue symbols are placentals.

There are clearly different slopes among species of marsupial and placental carnivores when over-eruption is regressed on body size (skull length) (interaction between body size and skull length: *F*_10, 244_ = 18.27, *p* < 0.0001; museum specimens) (table 1 and figure 3). All marsupial carnivore species show more pronounced over-eruption, as indicated by higher slopes, than any placental carnivore species across the whole range of body sizes measured. Over-eruption significantly increased with body size for Tasmanian devils but not in other species (table 1).

### (b) Canine tooth dimensions increase with body size and age

**Figure 4.**
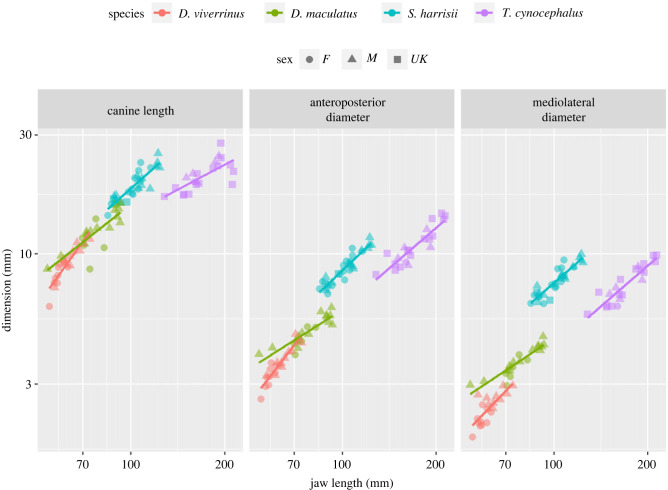
Changes in three canine dimensions (length, anteroposterior and mediolateral diameter) with increasing body size (jaw length) in four wild-captured Australian marsupial carnivores from museum collections.

In all four species of marsupial carnivores measured in museum collections, canine tooth size increased with body size (jaw length) in three dimensions (total length, anteroposterior and mediolateral diameters) (figure 4). The linear trends of these relationships varied among species for canine length and anteroposterior diameter with less evidence for differences among species for mediolateral diameter (table 2). Eastern quolls (intercept) had the steepest increase in canine size with log(jaw length) on all three measures. For canine length, the slopes were a bit shallower for Tasmanian devils, more so for spotted-tailed quolls and much shallower for thylacines. For anteroposterior diameter, the slopes were a bit shallower for Tasmanian devils, more so for thylacines and much shallower for spotted-tailed quolls. For mediolateral diameter, spotted-tailed quolls trended towards a shallower slope, although overall the slopes were not different (table 2 and figure 4).

**Table 2.** Parameter estimates of the differences among species (species by body size interaction) in the slope of the relationship between canine tooth dimensions (length, anteroposterior and mediolateral diameters) and body size (jaw length) for museum specimens of four species of marsupial carnivores. s.e., standard error; *t*, t test; *p*, *p*-value.

measure	species	slope	s.e.	t	*p-*value
canine length	*D. viverrinus* (intercept)	1.745	0.241	7.239	<0.001
	*D. maculatus*	−0.758	0.274	−2.767	0.007
	*S. harrisii*	−0.635	0.277	−2.295	0.024
	*T. cynocephalus*	−1.079	0.274	−3.945	<0.001
anteroposterior diameter	*D. viverrinus* (intercept)	1.616	0.180	8.991	<0.001
	*D. maculatus*	−0.823	0.205	−4.028	<0.001
	*S. harrisii*	−0.428	0.206	−2.075	0.041
	*T. cynocephalus*	−0.538	0.204	−2.638	0.01
mediolateral diameter	*D. viverrinus* (intercept)	1.298	0.196	6.624	<0.001
	*D. maculatus*	−0.464	0.223	−2.082	0.041
	*S. harrisii*	−0.206	0.225	−0.917	0.362
	*T. cynocephalus*	−0.213	0.222	−0.956	0.342

In wild marsupial carnivores canine tooth size increased through time in three dimensions (total length TH, anteroposterior APD and mediolateral MLD diameters) (TH: LRT = 12.67, *p* < 0.0001; APD: LRT = 16.09, *p* < 0.0001; MLD: LRT = 22.78, *p* < 0.0001) but with no effect of the animal's sex (TH: LRT = 0.24, *p* = 0.62; APD: LRT = 0.56, *p* = 0.46; MLD: LRT = −0.42, *p* = 1.00) (electronic supplementary material, figure S1). There was variation among species and individuals, with pronounced increases in the single spotted-tailed quoll and most Tasmanian devils and little or no changes on all dimensions in eastern quolls, and on some dimensions in Tasmanian devils. For the spotted-tailed quoll and the Tasmanian devils, the individuals whose canine teeth increased in length and the two diameters were independent juveniles at the first capture and were growing in body size over the time period of the measurements. The total age since birth and months since weaning for these individuals at the commencement of measurements were: the spotted-tailed quoll (SQ7–7/4 months) and Tasmanian devils: TD117 (11/2 m), TD120 (13/4 m), TD54 (11/2 m), TD59 (11/2 m), TD65 (11/2 m), TD69 (11/2 m) and TD75 (13/4 m). Note that time since birth in marsupial carnivores includes time spent in the pouch following a three-week gestation, and time in a den. Other Tasmanian devils were already mature or old at the commencement of the study and had completed growth, examples being TD15 and TD45 (both age 3 years at first measurement). In these two older individuals, while canine length did not change or decreased slightly over time, the two canine diameters continued to increase. While the canine teeth continue to over-erupt with age and the conical tooth becomes wider, tooth wear is an ongoing process eroding the tip of the tooth that reduces the total length of the tooth in old animals (figure 2).

### (c) Over-eruption of molar teeth

**Figure 5.**
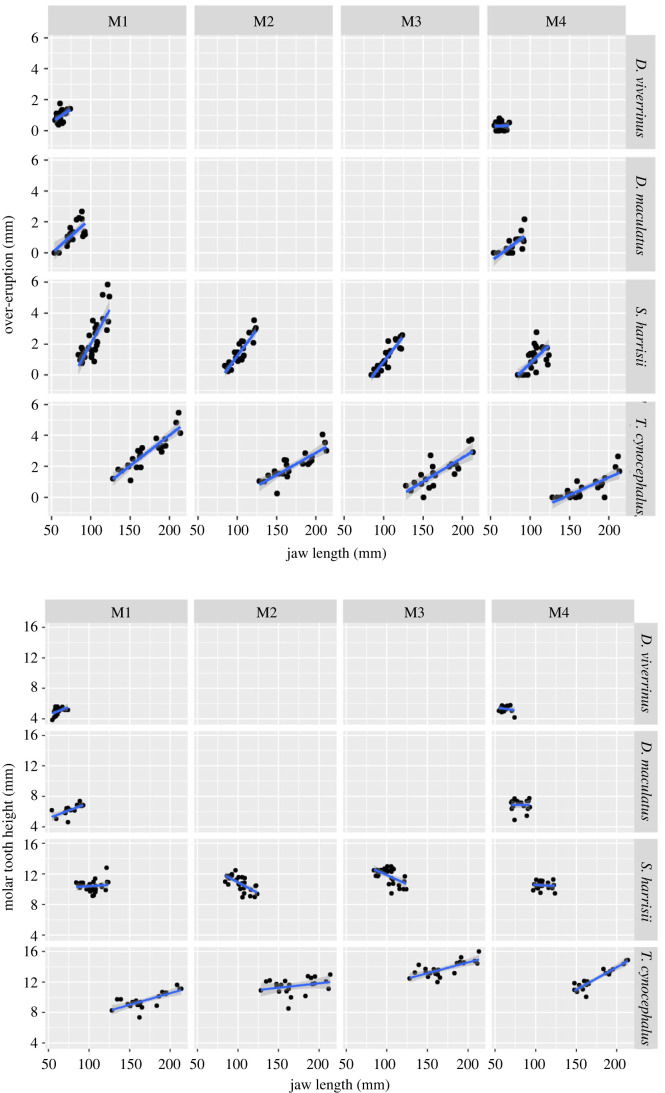
Extent of (*a*) over-eruption and (*b*) total tooth height across the molar tooth row, from M1 to M4, regressed against jaw length in four species of Australian marsupial carnivores from museum collections.

The extent of over-eruption in each molar tooth (M1 to M4) by body size (jaw length) was described by a linear trend in museum specimens of all four species of marsupial carnivores for which there were sufficient sample sizes (figure 5*a*). The interaction term testing for differences in these slope coefficients across the molar tooth row (M1 to M4) indicated that these within-tooth linear trends became less steep across the tooth row. In all four species, over-eruption was more pronounced in M1 and least in M4. This pattern was highly significant for devils, significant for thylacines, approaching significance for eastern quolls and not significant for spotted-tailed quolls (table 3*a*). This means that for devils and thylacines, as the individual grows the amount of over-eruption is highest in M1 and sequentially less for M2, M3 and M4, and this happens in a linear fashion (figure 5*a*). This shows that in these larger species, over-eruption in the molar teeth scales with body size according to the eruption sequence. The teeth that erupt prior to weaning when the animal is small show a lot of over-eruption between the time of their eruption and when the individual reaches adult size. This phenomenon becomes progressively less along the molar tooth row so that M4, which erupts five months after weaning in Tasmanian devils, shows little over-eruption.

The trend in molar tooth height with increasing body size (jaw length) in museum specimens was best described by a quadratic function for thylacines and Tasmanian devils. The trend was, by definition, linear for eastern and spotted-tailed quolls because only two teeth were measured (M1 and M4). In thylacines, molar tooth height increased significantly with body size (jaw length; which reflects increasing age) for all four molar teeth (figure 5*b*). The interaction between linear trends for each tooth was significant and positive, with later erupting molar teeth taller and increasing more in height than earlier erupting teeth (table 3*b* and figure 5*b*). The greatest increase in height with body size (jaw length) was in M4, then in M1 and M3, and least in M2 (figure 5*b*). In Tasmanian devils, the interaction among these slope coefficients along the molar tooth row (M1 to M4) was not significant (table 3*b*), indicating that molar height does not increase as the animal gets larger and older; indeed M2 and M3 decrease in height (figure 5*b*). The interaction between the linear trend in molar height with increasing body size between M1 and M4 was significant for eastern quolls and approaching significance for spotted-tailed quolls (table 3*b*). In both species, molar height increased with size and age in M1 but not in M4 (table 3*b* and figure 5*b*), according to the eruption sequence.

### (d) Composite patterns of over-eruption and tooth height in relation to tooth wear

**Figure 6.**
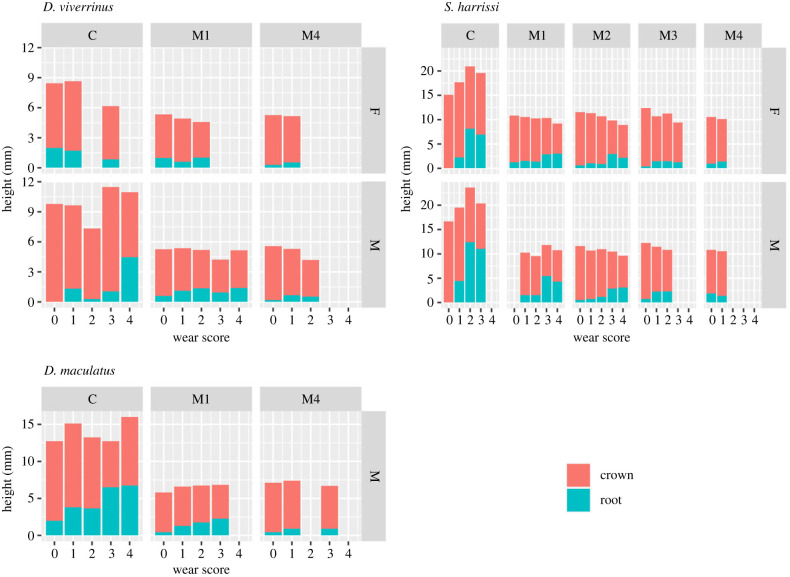
Canine and molar tooth height for each tooth wear stage in three species of marsupial carnivores, wild-caught at Cradle Mountain National Park in Tasmania, Australia. The stacked bars separate the components of total tooth height that comprise the enamelled crown and the exposed root where the tooth has over-erupted. Tooth types: canine (C), molars anterior to posterior: M1 to M4. Wear stages range from 0 (no wear) to 5 (advanced wear). F, female; M, male. There were too few data to analyse female spotted-tailed quolls.

Figure 6 shows the composite patterns of total tooth height, including the contribution of over-eruption, with increasing tooth wear for canine and molar teeth in repeatedly measured individuals of wild-living Tasmanian devils, spotted-tailed quolls and eastern quolls. For all three species, these stacked bar graphs show a general pattern of increasing canine length but stable or slightly decreasing molar height with wear stage. In both canine and molar teeth, there is an increasing contribution to total tooth height from over-eruption with increasing tooth wear. When the same measures were plotted for museum specimens, these patterns were not evident (electronic supplementary material, figure S2). The reason for this difference is probably that each data point in the museum dataset represented a different individual, compared with repeated measures over time of the same individuals in the wild dataset. Tooth wear and over-eruption are expected to increase with time and age. The wild dataset will reflect this temporal component. The museum dataset reflects snapshots in time.

## 4. Discussion

Over-eruption of the canine teeth is evident in placental carnivores, but it is more extreme in marsupial carnivores such that in some, presumably very old individuals with advanced tooth wear, the enamel cervical margin has progressed to near the tip of the canine. This suggests that canine teeth in marsupial carnivores erupt continuously through life. While dynamic erosion of the alveolar bone at the crest of where the canine tooth emerges from the maxilla may explain some of the variation in the increasing height of the canine tooth with age, the marked differences between marsupial and placental carnivores as well as extreme over-eruption in old marsupial carnivores indicate this is not a general explanation. The phenomenon of canine over-eruption is not restricted to the dasyuromorph marsupial carnivores. Continuous growth of canine teeth well into adult life or throughout life has been reported in extinct South American marsupial carnivore lineages in the Order Sparassidonta, including the ‘dog-like' borhyaenids (Family Borhyaenoidea) [21,22], and the marsupial saber-tooth carnivores (Family Thylacosmilidae) [23]. In these species, however, the canine teeth are open-rooted throughout life, which is not the case in dasyuromorph marsupials.

Carnivores need to have a functional set of teeth by the time they are weaned; canine teeth to kill and process prey and molar teeth with carnassial blade function to slice and consume meat. If over-eruption in marsupial carnivores is a compensatory response for tooth size limits imposed by monophyodont tooth replacement, the single generation of canine teeth should be small and scaled to the size of the jaw (body size) at weaning and increase in size as the individual grows to adult size. Juvenile devils and quolls possess a fully erupted set of anterior teeth at independence (weaning), when they are only a quarter to a third of their final adult size (approximate body weights at weaning and adult female:male: eastern quoll—0.35, 1.0 : 1.5 kg; spotted-tailed quoll—1.0, 2.5 : 4.0 kg; devil—2.5, 7.5 : 10.0 kg) [M Jones 1991, unpublished data and 24]. At weaning, the erupted tooth crown is completely enamelled, with the enamel cervical margin lying just within the gum (figures 1 and 2). These single generation anterior teeth, and to a lesser extent the molar teeth, over-erupt as the individual grows, increasing in length and diameter. Over-erupted teeth comprise the enamelled tooth crown, which becomes smaller as the tip wears, and an increasing extent of exposed root not protected with enamel (figures 1 and 2). By contrast, placental carnivores have a fully erupted small set of first generation anterior teeth at weaning age and are nearly full grown when these are replaced by their larger and permanent second generation teeth, both being scaled to body size [4].

### (a) Consequences of over-eruption

Over-eruption of canine teeth in marsupial carnivores results in a net increase in height and diameter as the individual grows to adult size, with the greatest rate of increase coinciding with the period of rapid growth from small juvenile at independence to adult size. The weaned juvenile has small canine teeth scaled to its smaller jaw and the fully grown adult has large canine teeth scaled to its size. Carnivore canine teeth are tapered conically at both ends, with maximal width half-way along the length (figures 1 and 2; electronic supplementary material, figure S3). The conical shape of the distal half of the canine tooth results in a gradual increase in both the length and width of the canine teeth with over-eruption. Presumably even in very old animals, the proximal half of the tooth, which diminishes in diameter, remains within the skull or jaw. The rate of increase in canine dimensions is generally scaled with body size of the species, being greatest in the smallest species, the eastern quoll, and least in the thylacine. The devil is an exception to this pattern and the greater rate of over-eruption with size may relate to the high degree of tooth wear experienced in this specialist osteophage or bone-eating species [2].

In general, the canine teeth increase less in the mediolateral than the anteroposterior diameter, reflecting species differences in canine shape and killing function [9]. Spotted-tailed quolls, which kill relatively large prey with a penetrating bite, have the most laterally compressed canine teeth, narrow in the mediolateral and broader in anteroposterior diameter, and show the least increase in mediolateral diameter with body size [2,10]. Eastern quolls and thylacines also have laterally compressed canines, reflecting a penetrating killing bite of prey small relative to body size in the largely insectivorous eastern quolls and in thylacines [2,10,25]. The devil is again the exception, with canines that are rounded in cross section, an adaptation to bone eating that reduces the chance of canine fracture [2,9,11,16]. Regardless of species differences in killing behaviour, concurrent increase in canine tooth diameter with increasing tooth height is important to retain canine tooth strength.

The molar teeth in marsupial carnivores also over-erupt, generally increasing in height as the individual grows to adult size, but to a much lesser extent than in the canine teeth. Molar over-eruption is positively scaled to body size, being most evident in the two larger species, the thylacine and the devil, and minimal in the two smaller species, the quolls. The lifelong patterns of over-eruption across the molar tooth row vary among species, reflecting a combination of over-eruption and tooth wear, and thus species trophic ecology. The contribution of over-eruption to total tooth height is greater in the anterior molars which erupt first (M1, M2) than in the posterior molars which are last to erupt (M3, M4).

### (b) Occlusal pressure as a mechanism of over-eruption

In mammalian species with closed-rooted teeth, over-eruption or continuous eruption of teeth is reported to occur in response both to tooth wear [13] and to the loss of the opposing tooth of an occlusal pair [26]. In adult wild great apes, for example, continual eruption means that total tooth height does not vary with wear stage or age [13]. Because molar teeth have complete occlusion, over-eruption has a clear mechanistic role in compensating for changes in tooth height in response to tooth wear (the amount of enamelled crown that has been worn away) and to the loss of an occlusal partner. The latter is observed occasionally in the canine and molar teeth of live devils and quolls (personal observation), with canine fracture reasonably common but molar tooth fracture rare in these marsupial carnivores [10]. I propose a third related mechanism in marsupial carnivores, that as the animal grows and its skull size increases, the distance between the maxilla and mandible enlarges slightly, functioning to deliver a slow release in occlusal pressure between paired teeth (figure 1; electronic supplementary material, figure S3).

In the canine teeth of marsupial carnivores, the major mechanism driving over-eruption is possibly the gradual release of oblique occlusal pressure as the animal grows, with dynamic tooth wear facets on the tip and antero and posterior edges of the tooth in some individuals contributing to the changing occlusal pressure. While the canine teeth do not have the complete occlusion seen in the molar teeth, the upper and lower canines touch, sliding obliquely against each other during jaw movement. As the animal grows, the increasing skull size including a larger and longer mandibular condyle may create a greater distance between the jaws (figures 1 and 2; electronic supplementary material, figure S3) which gradually releases occlusal pressure. This would trigger a natural physiological response of over-eruption [14], which combined with the conical shaped canine tooth results in an increasing canine tooth diameter and length which then functions to maintain occlusal pressure. This phenomenon is suggested also by the decreasing anterior to posterior pattern of over-eruption scaled to the age and body size at which canine and molar teeth erupt (figure 1). The canine teeth in marsupial carnivores continue to over-erupt throughout life, suggesting primacy of a mechanism of low occlusal pressure although tooth wear also plays an important role.

The amount of molar over-eruption in marsupial carnivores is scaled with body size and therefore skull size and inter-jaw distance, both among species and during ontogeny. Over-eruption is most evident in the two larger species, the thylacine and the devil, and minimal in the two smaller species, the quolls. The extent of molar over-eruption is greatest in the anterior M1 and decreases to least in the posterior M4 in all marsupial carnivore species measured, correlating with the eruption sequence [5] relative to age and size of the individual. The first two molars (M1, M2) erupt into a very small juvenile jaw prior to weaning and will be exposed to a greater increase in inter-jaw distance than M3 and M4 which erupt by six months after weaning when the animal is two-thirds grown.

### (c) Tooth wear as a mechanism of over-eruption

The significant contribution of tooth wear to over-eruption is suggested by the increasing amount of over-eruption with increasing canine tooth wear scores, and to a lesser extent molar tooth wear scores, in wild marsupial carnivores. This is evident in both devils, which as specialized osteophages have a high-wear diet throughout life, and in quolls which consume the bones only of small prey. Tooth wear, and the physiological response to tooth wear of over-eruption (electronic supplementary material, figure S3), occur continuously through life, unlike growth which is complete early in independent life (by 3 years of age in male devils, and 2 years in female devils and quolls). In advanced cases of tooth wear, canine and molar tooth height typically declines. In very old devils, the extent of canine and anterior molar tooth wear is such that the enamel cervical margin is near the tip of the tooth, with most of the crown having worn away, and in rare cases the teeth can be worn almost to the gum. Canines in very old animals are prone to fracture [2], particularly as lateral wear facets reduce tooth diameter and thus structural strength.

Molar tooth wear is related to tooth function, being minimal in the carnassial teeth which need to stay sharp to slice meat effectively, and high in the bone-cracking teeth of specialized osteophages [27]. The carnassial teeth are positioned well back in the jaw in both placental and marsupial carnivores, at a similar biomechanical position of 50% along the jaw length [2,4,16] and remain sharp during life in most species. While placental carnivores have a specialized carnassial tooth (M1), in marsupial carnivores each molar tooth has a carnassial blade and functions in meat slicing sequentially as the animal grows, with M4 and M3 having primary carnassial function in adult life [2,4,16]. Bone is processed further forward in the jaw, on P3 (premolar) in placental hyaenas [16] and M2 in marsupial devils [2], the two specialist osteophages in this study. This explains why M2 and the adjacent M1 and M3 in devils show extensive wear during life, typically decreasing in height. Even small devils eat bone and tooth wear on M2 is evident in the first subadult year of independent life. By contrast, thylacine skulls have low wear scores on all teeth, with no individuals with a wear score on M4 greater than zero. Thylacines lack bone-eating specializations and are thought to have killed prey small relative to their body size [2,10,25,28].

### (d) Future directions and conclusions

An unexplored potential consequence of tooth over-eruption is the abrasion strength of the exposed section of tooth beyond the enamelled proximal crown. Enamel structure is strongly correlated with tooth function and enamel microwear on teeth is attributed to mechanical stresses of shearing and compression [29]. In the larger marsupial carnivore species, devils and thylacines, but not in quolls, the premolar and molar teeth have greater diversity of enamel types than the incisors and canines, reflecting their function in shearing and grinding rather than grabbing and holding prey [30]. In devils, compared with thylacines, there is less differentiation in enamel structure between the premolar and molar teeth, probably reflecting adaptation to their bone-eating diet [30]. Placental osteophagous species, e.g. hyaenas, also have a more complex enamel structure [31]. It is not clear whether the structural integrity of the exposed over-erupted tooth section that lacks enamel is compromised. No damage was observed to the exposed root on any individuals, live or museum specimens. Microwear on the teeth might be primarily on the leading edges of the canines and the molars [29], which remain protected by enamel.

This study addresses an unresolved question in understanding the consequences of different ontogenetic patterns of tooth replacement in mammals, how the anterior teeth in monophyodont marsupials, which are fully erupted at weaning when the animal needs functional dentition, scale to the jaw size of both the small juvenile and the adult [3]. Analysis of the ontogeny of changes in canine and molar tooth size, tooth wear and eruption among the extant and recently extinct species of Australian dasyuromorph marsupial carnivores, reveals a mechanism of tooth over-eruption that circumvents the potential ‘constraint’ of the monophyodont condition. Over-eruption, particularly of the conical-shaped canine teeth allows for a smaller canine to be fully functional in the jaw of a newly weaned juvenile and for the length and diameter of the tooth to increase as the animal grows to mature body size. The mechanisms that produce over-eruption in marsupial carnivore teeth appear similar to those operating in other mammals, being a consequence of tooth wear and loss of occlusal partner, with the variation that over-eruption may respond to the changing occlusal pressure as the distance between mandible and maxilla increases with growth in body size from juvenile to adult. Over-eruption in marsupial carnivores is substantially greater than in ecologically equivalent placental carnivores which have diphyodont tooth replacement. Future research could explore this phenomenon across a broader range of marsupial and placental mammals exhibiting different patterns of tooth replacement.

## Data Availability

Tooth and skull measurements from museum specimens and from live, wild animals: Dryad https://doi.org/10.5061/dryad.v6wwpzh19 (Jones 2023) [32]. Supplementary material is available online [33].
